# Towards virtual histology with X-ray grating interferometry

**DOI:** 10.1038/s41598-023-35854-6

**Published:** 2023-06-03

**Authors:** M. Polikarpov, J. Vila-Comamala, Z. Wang, A. Pereira, S. van Gogh, C. Gasser, K. Jefimovs, L. Romano, Z. Varga, K. Lång, M. Schmeltz, S. Tessarini, M. Rawlik, E. Jermann, S. Lewis, W. Yun, M. Stampanoni

**Affiliations:** 1 Swiss Light Source, Paul Scherrer Institut, 5232 Villigen-PSI, Switzerland; 2 Institute for Biomedical Engineering, ETH Zurich, 8092 Zurich, Switzerland; 3 Department of Pathology and Molecular Pathology, University Hospital Zürich, 8091 Zurich, Switzerland; 4 Department of Diagnostic Radiology, Translational Medicine, Lund University, Lund, Sweden; 5 Unilabs Mammography Unit, Skåne University Hospital, Malmö, Sweden; 6 EPFL, Lausanne, Switzerland; 7 Sigray Inc., Concord, CA 94520 USA; 8 Present Address: Department of Engineering Physics, Tsinghua University, Haidian District, Beijing, 100080 China

**Keywords:** Biophysics, Cancer, Health care, Medical research, Engineering, Optics and photonics, Physics

## Abstract

Breast cancer is the most common type of cancer worldwide. Diagnosing breast cancer relies on clinical examination, imaging and biopsy. A core-needle biopsy enables a morphological and biochemical characterization of the cancer and is considered the gold standard for breast cancer diagnosis. A histopathological examination uses high-resolution microscopes with outstanding contrast in the 2D plane, but the spatial resolution in the third, Z-direction, is reduced. In the present paper, we propose two high-resolution table-top systems for phase-contrast X-ray tomography of soft-tissue samples. The first system implements a classical Talbot–Lau interferometer and allows to perform ex-vivo imaging of human breast samples with a voxel size of 5.57 μm. The second system with a comparable voxel size relies on a Sigray MAAST X-ray source with structured anode. For the first time, we demonstrate the applicability of the latter to perform X-ray imaging of human breast specimens with ductal carcinoma in-situ. We assessed image quality of both setups and compared it to histology. We showed that both setups made it possible to target internal features of breast specimens with better resolution and contrast than previously achieved, demonstrating that grating-based phase-contrast X-ray CT could be a complementary tool for clinical histopathology.

## Introduction

Breast cancer is the most common type of cancer globally^1^. For this reason, the importance of timely preoperative diagnostics is of utmost relevance with mammography being the main screening technique, aiming at decreasing mortality rate worldwide^1–3^. As second-level examination in the clinical workflow, and if there is a suspicious finding on the mammogram, mammography is followed by ex-vivo diagnostics: typically, by CNB—core needle biopsy, or by VAB—vacuum assisted biopsy^4–6^. Histopathological analyses of the preoperative biopsies and the interdisciplinary discussion with radiologists, pathologists and gynecologists is the mandatory clinical workflow to decide which is the next diagnostic or surgical step for a given patient. During the breast core biopsy procedure, a sample is taken from the breast parenchyma, fixed in formaldehyde in most institutions, embedded in paraffin blocks and sectioned. It is followed by a subsequent microscopic examination by a certified pathologist, making it possible to reach a very high 2D resolution of 250–500 nm and outstanding contrast inside the tissue^7^. The first histopathological diagnosis can be reached within 6 h after biopsy removal, but, due to high demand in the clinical workflow, the assessment on average takes a few days. Covering the aforementioned requirements and providing a genuine 3D insight view of the investigated sample, high-resolution X-ray tomography can serve as an additional support tool to histology^8^. However, low contrast and resolution of commercially available scanners remain a limiting factor for routine *ex-vivo* clinical examination of breast tissues.

Conventional X-ray imaging is based on attenuation contrast which is inherently low within breast tissue^9^. Here, X-ray phase contrast^10^ is one of the promising modalities for soft tissue imaging^11^, in general, and breast imaging, in particular^12,13^, as it additionally enhances interfaces between materials with different refractive indices and electron densities. Therefore, it facilitates visualization of high-frequency details inside the specimen, especially when no additional contrast enhancements by absorbing agents is used. Phase-contrast imaging has become an established technique at synchrotrons—X-ray facilities with powerful and collimated radiation^14^. Due to the small size of the X-ray source, large source-to-sample distances, high collimation, flux, monochromaticity, and sensitive detection setups with effective pixel sizes down to several hundred of nanometers, it is possible to routinely make tomographic scans of biological samples with high contrast, image quality and submicron resolution^15–25^. In recent years, attempts have been made to bring phase-contrast imaging into a clinically compatible workflow. In particular, a first clinical study on phase-contrast mammography was conducted with synchrotron radiation, demonstrating superior visibility of breast abnormalities and pathological changes at dose levels lower or comparable to conventional mammography^12,26–28^. In another work^29^, high-resolution phase-contrast images of breast tissue specimens have been acquired and analyzed, demonstrating image quality and resolution close to histopathology. Authors^29^ discussed the possibility to use the method for numerous clinical applications such as intraoperative specimen assessment, tumor volume and disease extent assessment, with overall improvement in diagnostic accuracy. However, access to synchrotrons is not straightforward and it relies on a strict proposal-based peer-reviewed beamtime allocation mechanism. Therefore, the research on synchrotrons is problematic to be used for routine clinical work.

The development of edge-illumination techniques^30^, grating-based X-ray interferometry (GI)^31^ and GI-based imaging methods enabled successful implementation of phase-contrast^32^ and dark-field imaging^33^ on incoherent, commercially available low-brilliance X-ray sources. This brought new opportunities to translational imaging^34^, especially to human lung^35–37^ and human breast^38–42^ imaging, where first prototypes of GI-based investigational devices for in-vivo diagnostics were recently demonstrated^43,44^. It was also recently proposed^45^ to use GI-based phase-contrast imaging for ex-vivo tomography and better visualization of samples with ductal carcinoma in-situ (DCIS). The study has shown promising results, but the resolution of the system—about 100 µm—was not sufficient to compete with histological work-up. To address the aforementioned limitation, in the present paper we present a GI-based X-ray system for high-resolution phase-contrast tomography, that relies on classical Talbot–Lau interferometry and allows ex-vivo 3D tomography of unstained human breast samples in formalin.

In addition, we propose a second, slightly modified X-ray system, which operates with the Sigray MAAST X-ray source with a structured anode. This unique X-ray source was introduced to the market in 2021^46,47^ and holds a promising potential for soft-tissue imaging. It was shown that the implementation of such source relaxes the complexity of the classical Talbot–Lau system and allows to increase signal-to-noise ratio, improve uniformity of the FOV, decrease experimental time and improve overall user experience due to simpler setup operation. In the present paper, we for the first time demonstrate its applicability to soft-tissue X-ray imaging and, specifically, to imaging of human breast specimens with DCIS.

We use these two table-top tomography systems to investigate the feasibility of performing phase-contrast X-ray imaging of breast specimens, within the resolution range of 10–20 µm and at different acquisition times, comparing obtained results to conventional histopathology.

## Results

**Figure 1:**
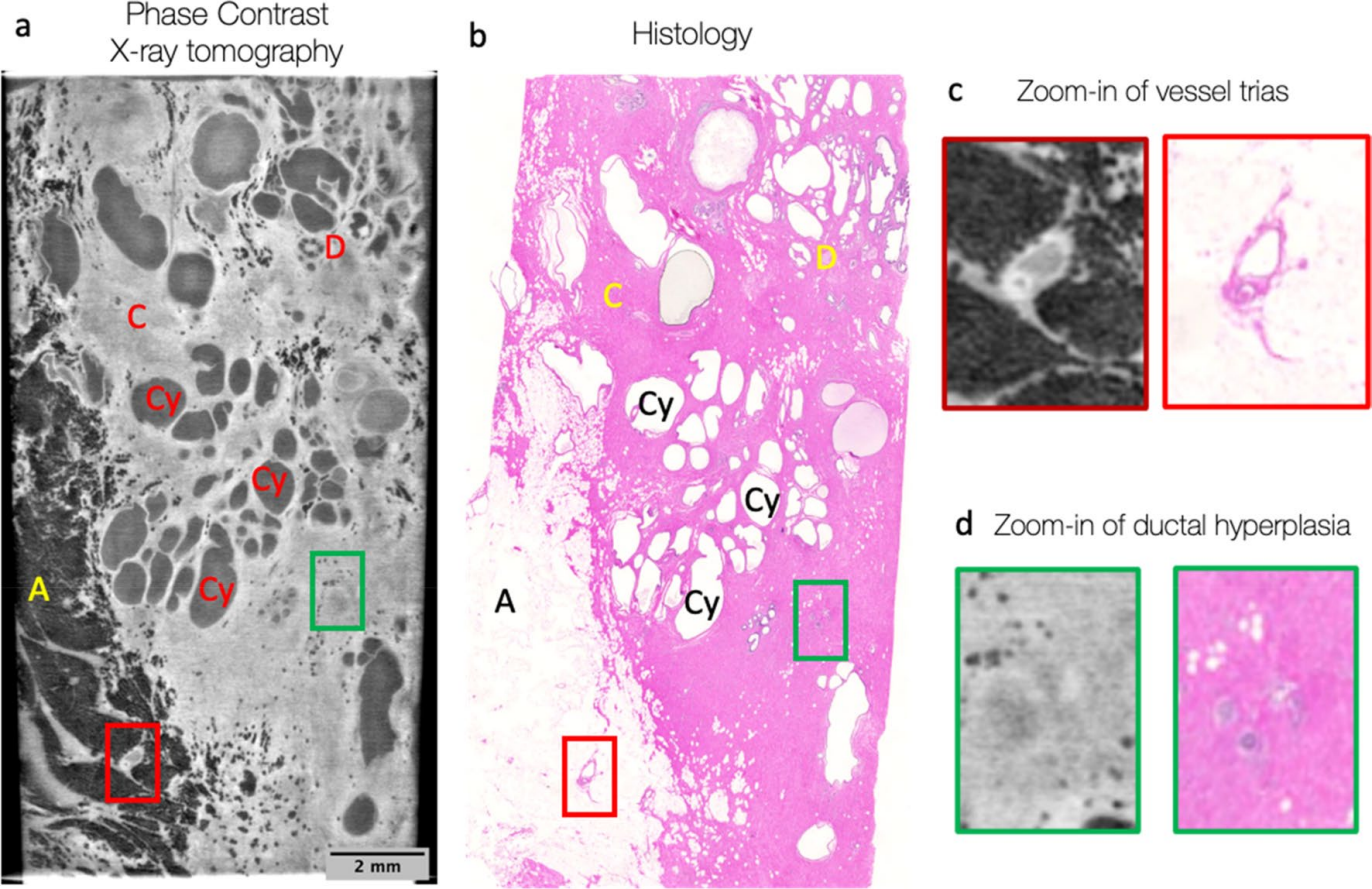
High-resolution phase-contrast imaging (TLI setup) and corresponding histopathological assessment of a human breast tissue (sample 1). (**a**) a coronal slice reveals details of the internal structure and allows to discriminate between: adipose tissue (fat cells, A—dark grey in **a** and white in **b**); connecting tissue (C—light grey in **a** and pink in **b**); milk ducts (D); multiple cysts (Cy); vessel trias (red square and **c**) and usual ductal hyperplasia (green square and **d**). This is confirmed by the corresponding histological slide (**b**) at the same sample orientation.

In a first experiment, a sample from the human breast in formalin (patient 1, sample 1) was scanned at the GI PC X-ray CT setup, named TLI and based on a Talbot–Lau interferometer, providing a voxel size of 5.57 µm, maximal field of view at the sample position of up to 2 × 2 × 2 cm^3^, angular sensitivity of 40 nrad and visibility of 19.5%. One coronal slice from the 3D volume was compared to the histological slice with the identical sample orientation (Fig. 1). The X-ray images were assessed by a radiologist and a pathologist, and no malignant features were identified. The measured (via Fourier Schell Correlation, FSC) resolution of the acquired X-ray data with this sample was 14 µm (see supplementary materials for more details) and coupled with high image contrast it allowed not only to clearly visualize multiple features in the X-ray image (fat, connecting tissue, vessels, cysts and milk ducts), but also to match the histological image. The histology revealed mastopathic changes, such as cysts, fibrosis, and apocrine metaplasia. No calcifications were visible neither on X-ray nor on histological slices.

**Figure 2:**
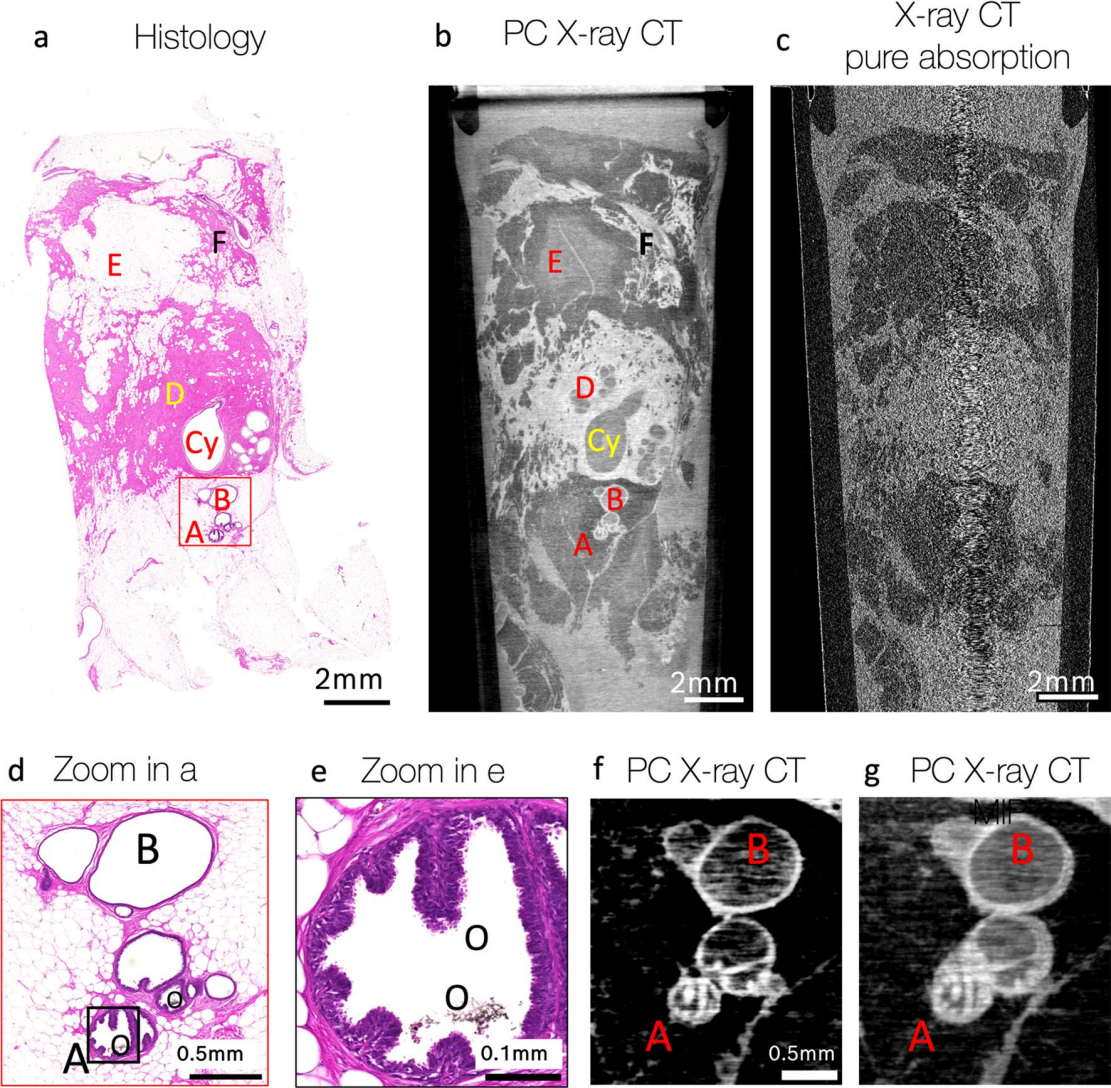
High-resolution phase-contrast imaging (MAAST setup) and corresponding histopathological assessment of a human breast tissue (sample 2). Histology (**a**) and GI phase-contrast X-ray CT (**b**) revealed numerous structures inside the tissue: milk duct with columnar cell metaplasia (A); dilated duct or duct ectasia (B), cysts (Cy), connective tissue with fibrosis (pink areas in **a** and white—in **b**; for example: D); fat cells (white areas in (**a**) and dark gray—in (**b**); for example: E); vessels (for example: next to F). (**c**)—Grating interferometer allows to reconstruct images with absorption, phase-contrast and dark-field signals from one dataset. The corresponding coronal slice in absorption CT as a comparison, showing poor contrast and an obviously lower image quality. (**d**)—Zoomed-in red square selection in (**a**). (**e**)—zoomed-in black selection in (**d**). Higher magnification on (**d**–**e**) allows to see oxalate crystals inside the milk duct (O), which are also visible as bright spots on the single X-ray CT slice (**f**, increased contrast compared to **b**–**c**, **g**) and its Maximum Intensity Projection over 20 slices (**g**).

**Figure 3:**
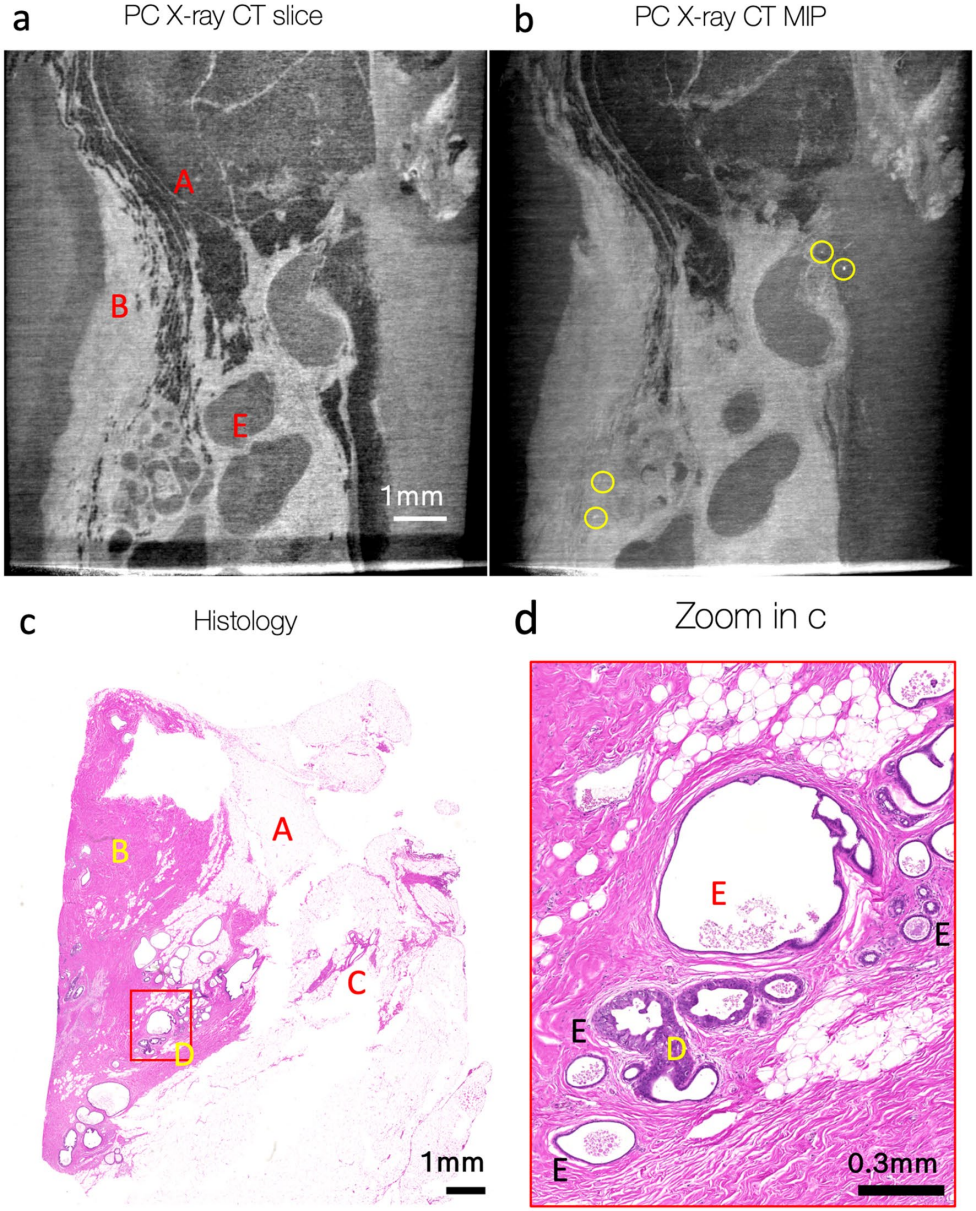
Lower part of sample 2 (with respect to the Fig. 2, **b**), examined with histology and GI phase-contrast X-ray CT (MAAST setup). X-ray CT (**a**–**b**) and histological (**c**–**d**) slices are not fully mutually aligned. (**a**)— one slice in the tomographic reconstruction of the data acquired with MAAST X-ray setup. It reveals some features, that can be also identified at the histological cross-section (**c**): fat (A), connecting tissue (B), vessels (C), benign apocrine cysts (E). (**b**)—Maximum Intensity Projection over 50 slices of the tomogram in (**a**) helps to notice bright white spots with different intensities that are likely calcifications. (**d**)—zoomed-in area at the histological slide (red square, **c**) highlights benign secretory calcifications inside cysts (E), as well as several duct cross-sections with low-grade DCIS.

In another experiment, the second GI PC X-ray CT setup, named MAAST and implementing the X-ray source with a structured anode, was used to scan the sample of the same human breast (patient 1, sample 2). The setup reached an angular sensitivity of 62 nrad, visibility of 16%, voxel size of 8.52 µm, and the maximal field of view at the sample position of up to 3.5 × 3.5 × 3.5 cm^3^. The measured resolution (via FSC) of the setup with the samples was measured to be 18 µm (see supplementary materials for more details). Like the assessment of Sample 1 with TLI, one coronal slice from Sample 2 was compared to the histological slice with an identical sample orientation (Fig. 2). Various sample features were identified in both X-ray and histological images, such as milk ducts, cysts, fat, connecting tissue, vessels. Specifically, the milk duct with columnar cell metaplasia (Fig. 2a–b, feature A)—because it contained oxalate crystals inside (Fig. 2d–e, features O)—resulted in high contrast with bright, white areas in the X-ray images (Fig. 2f–g). The combination of fibrosis, cysts and columnar cell metaplasia was an indication of benign mastopathy. Even though no obvious malignant features were identified in these specific X-ray and histological slices, a more detailed investigation of the X-ray data detected small calcifications, spread through the volume in the lower part of the sample (Fig. 3a–b). Calcifications, observed in the X-ray CT, were likely extraductal since no ductal structures were visible. Guided by these findings, a detailed histological investigation of this specific region was performed, revealing not only benign secretory calcifications inside cysts (Fig. 3c–d, features E), but also a few ductular cross sections with a low-grade DCIS.

**Figure 4:**
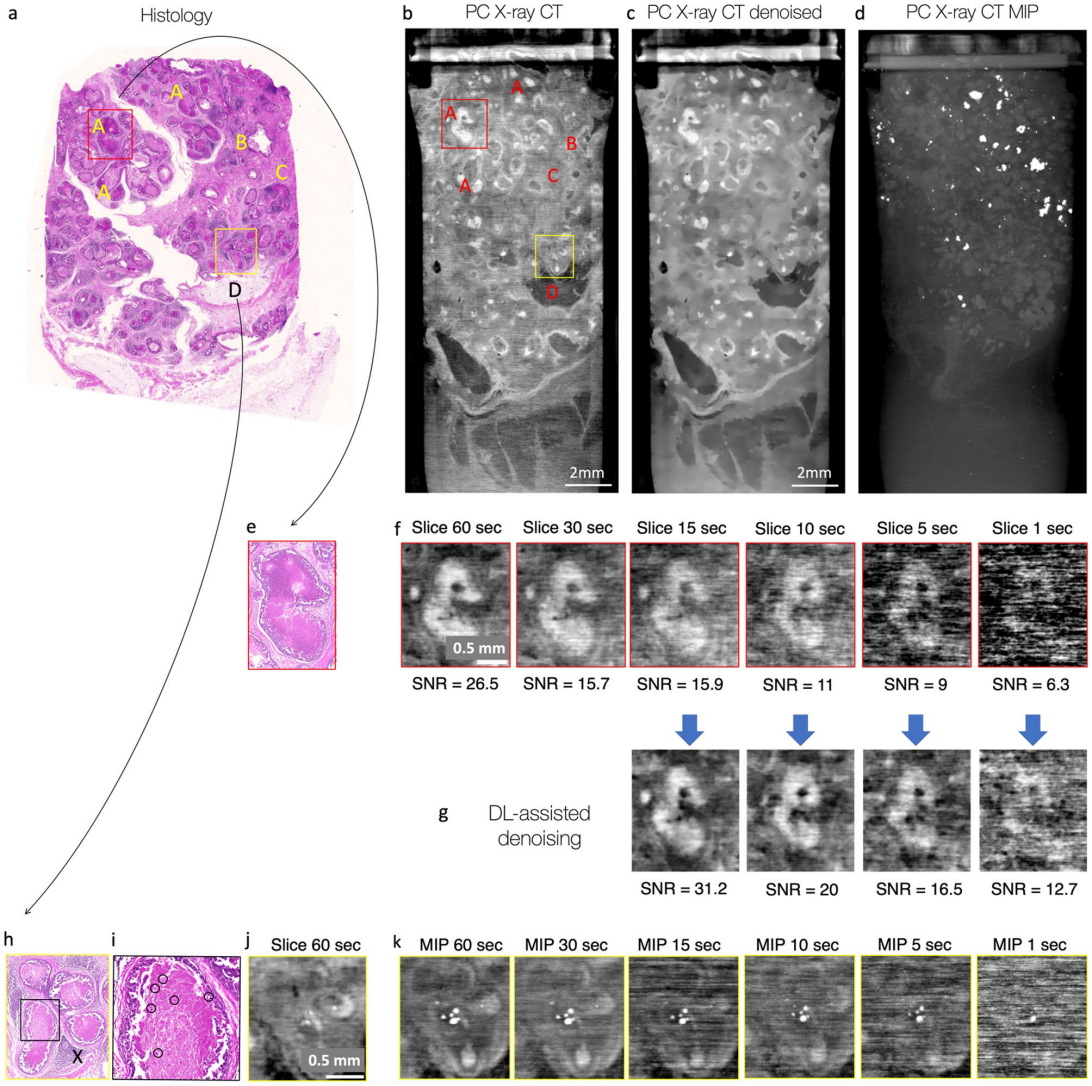
Sample 3 with high-grade DCIS was examined with histology and GI phase-contrast X-ray CT (MAAST setup). (**a**)—Histological examination reveals numerous malignant cells inside milk ducts (A). Less affected ducts (B), fat (D) and fibrosis (pink area in the connecting tissue; for example, C) are also visible. (**b**)—One tomographic slice of the data acquired with MAAST X-ray setup reveals most of the features, identified at the histological cross-section. Absorbing areas inside the necrotic ducts (A) are clearly distinguishable. (**c**)—denoised version of the image *b* allows to better see the shapes of individual ducts. A non-local Means 2D filter was applied with smoothing factor 2 and auto-estimated sigma. (**d**)—Maximum Intensity Projection over 1000 slices of the tomogram at (**b**). The image reveals numerous calcification clusters, that are an indication of the severe necrosis inside milk ducts. (**e**)—zoomed-in yellow square from the histological slice (**a**) highlights necroses inside the milk duct associated to DCIS. (**f**)—while necrotic formations inside the duct are also nicely visible with X-ray CT, the contrast is highly dependent on the exposure time per image in a given CT scan. (**g**) Deep-learning Noise2Noise-based denoising allows to clearly visualize inhomogeneities inside the milk duct with three times decreased acquisition time: the image quality of denoised 10-s image is comparable with, and even superior to the quality of the original 30-s one (SNR of 20.4 vs 15.7). (**h**) zoomed-in red square from the histological slice (**a**) highlights three milk ducts with severe necrosis. Milk ducts are surrounded by areas with inflammation (X). (**i**)—zoomed-in area from (**h**) reveals small calcifications that grow on the necrotic formations (yellow circles), they are also visible as white saturated dots at the X-ray CT slice (**j**). (**k**) Maximum Intensity Projections over 20 slices help to highlight calcifications inside ducts more prominently—calcifications remain visible even when the contrast degrades while decreasing the exposure time per image in the CT scan.

Finally, sample 3 (patient 2) with high-grade DCIS was scanned with the MAAST setup and compared to the microscopic histological image (Fig. 4). Malignant cells inside breast ductalar structures were visible with both modalities (Fig. 4a–c). Necroses inside DCIS-affected milk ducts were also seen very prominently as bright white spots in MIP (maximum intensity projection) X-ray data (Fig. 4d). Further zoom-in into the DCIS-affected ducts (Fig. 4e–f, h–j) allowed to compare the visibility of DCIS, necroses with calcifications between histological and X-ray images. DCIS was highly visible when operated with the highest exposure of 60 s per image (~ 65 h for one full CT), while contrast decreased significantly with the reduction of the exposure time. For the acquisition with less than 10 s per image (~ 11 h per one full CT) it was not possible to detect DCIS. However, a very bright signal from DCIS-related calcifications remained visible in MIP data (Fig. 4k) even when the exposure time was decreased down to 1 s per image (~ 1 h per full CT), thus indicating areas that might be of potential interest for a follow-up histological assessment. To enhance the image quality, we also explored the feasibility of deep learning-based denoising of our µCT data. The results in (Fig. 4g) show that data-driven denoising allows to reduce the exposure time by more than a factor 3 without sacrificing image quality. In fact, the signal-to-noise ratio of the images obtained with an exposure time of 30 s was 15.7, while the denoised 10-s exposure data had a signal-to-noise ratio of 20.4. In general, the data-driven denoising pipeline allowed to increase the signal-to-noise ratio by a factor of two across all exposure times (1–15 s).

## Discussion

In this work we have demonstrated the potential of high-resolution grating-interferometry phase-contrast X-ray tomography (GI PC X-ray CT) of formalin-fixed human breast specimens. Our results show that the proposed systems could be a valuable addition to the pre-operative imaging analyses. They could allow to tailor histopathological data collection and to ultimately increase its throughput, due to GI PC-systems’ ability to visualize inner interfaces and micro-calcifications with high resolution and contrast across several dose/acquisition modalities.

We have introduced a GI PC X-ray lab-CT system (TLI) which, with a voxel size of 5.57 µm, yielded high-contrast ex-vivo images of a human breast specimen (sample 1) with an angular sensitivity of 40 nrad, and a measured resolution of 14 μm (defined by the FSC), which is better than previously published. We were able to demonstrate that a TLI setup could successfully visualize inner details (adipose tissue, connecting tissue, milk ducts, cysts, and vessels) of a benign fixed human breast specimen and identified structures were correlated with the microscopic histological assessment.

Moreover, to reduce the mechanical complexity of the TLI-system, we have for the first time demonstrated the applicability of an X-ray source with a structured anode (Sigray MAAST) for high-resolution phase-contrast X-ray imaging of breast specimens. The implementation of the imaging system in combination with the MAAST structured anode also allowed us to achieve high image quality, contrast, and resolution. A voxel size of 8.52 µm, with a measured resolution of 18 µm (defined by the FSC), combined with a high angular sensitivity of 62 nrad and a field of view of 3.5 × 3.5 cm^2^, enabled the ex-vivo assessment of structural features inside two breast samples with low-grade DCIS (sample 2) and high-grade DCIS (sample 3), respectively. Apart from the high-resolution information about the structures, we were able to detect microcalcifications in both samples—this will be discussed in more details in the next paragraph. It is important to mention now, however, that microcalcifications have provided a guidance to further histological assessments of the sample 2: highlighting areas of potentially suspected malignancies in 3D on a tomogram, they tailored the field-of-view of the pathological examination, leading to the better detection of areas with DCIS.

We would like to discuss in more details an opportunity of clinical guidance for histology that GI phase-contrast X-ray imaging provides. In our experiment, to ease the detectability of the malignant intraductal formations in the X-ray images, we used a Maximum Intensity Projection (MIP). It provided prominent visualization of the micro-calcifications that might be related to severe necrotic clusters, often encountered in non-invasive breast cancer such as DCIS. Although breast calcifications are usually non-cancerous (benign), certain patterns of calcifications—such as tight clusters with irregular shapes and fine linear appearance—may indicate malignancy. For instance, in the sample 3 with high-grade DCIS, micro-calcifications appeared as brighter clustered spots on the individual tomographic slices and became even more prominent when being stacked over several slices. Related calcifications were most likely visible during the prior in-vivo examinations (incl. mammography) and were one of the triggers for the surgical intervention. However, the added value of the GI PC X-ray lab-CT lies in the potential support in the preoperative diagnostic setting, providing additional imaging information prior to the histopathological examination. In these scenarios, histopathology serves as a main tool for a diagnostic assessment of biopsies with the diameter of few millimeters, that are smaller than the cm-sized surgical breast samples, assessed in the present study. As an addition, GI PC X-ray lab-CT could help to tailor such assessment: making a 3D-CT of multiple (for example, 20) mm-size specimens at once could help to detect micro-calcifications’ distribution and shape in some of those. Different samples would be bundled together on the sample stage of the MAAST setup, which has the field of view of up to 3.5 × 3.5 × 3.5 cm^3^, however a 3D nature of acquired CT data would still allow to clearly distinguish features inside of them. As a result, this would lead to a tailored histopathological assessment with a pre-selection of interesting samples to undergo biopsy.

Acquisition time (or dose) affects contrast and resolution and is highly related to clinical utility. High resolution and contrast come at the expense of dose, and in the present study we have achieved the highest resolution and contrast (Fig. 4) with an acquisition time of 60 s per image (~ 65 h per full CT) which may be just too long for a daily clinical operation. Therefore, images with different acquisition times (1–60 s per image) were also compared in Fig. 4. We have noticed that the diagnostically relevant features still could be recognized on the tomograms taken with the acquisition time of 15–30 s per image, which was also confirmed by the SNR measurements. Moreover, we have shown that deep learning-based denoising helped to boost contrast of a 10-s image up to the quality of a 30-s image, which enables an additional reduction in total acquisition time: ~ 11 h per one full CT with 10-s exposure. As expected, the contrast degraded significantly when we further reduced the exposure times down to less than 10 s per image. However, the very bright signal from calcifications remained partially visible even when the exposure time was decreased down to 1 s per image (~ 1 h per full CT), thus still highlighting some areas that were of potential interest for further histological assessment.

We would like to highlight the proposed image quality enhancement with the help of deep learning, as it can be extremely valuable in the field of phase-contrast X-ray imaging in general and is technically not limited to grating interferometry only, but can be applied to other X-ray imaging techniques (e.g. EI).

Further technical enhancements of our systems to reduce acquisition time can be approached through various avenues. Currently, an immediate reduction in acquisition time can be achieved by twofold binning of detector pixels and a four-fold decrease in the number of acquired projections. As depicted in supplementary Fig. S7, the reduction in acquired projections will result in a 20–25% decrease in signal-to-noise ratio (SNR) due to increased image noise. Conversely, binning will enhance SNR by 5%. Consequently, employing both measures simultaneously, would decrease SNR by 20% but it would allow for at least a four-fold improvement in acquisition time and a corresponding reduction in absorbed dose. Such approach would lead to the decrease of resolution, however the high tissue contrast would still remain, providing an added value to pathologist. Additionally, the improvement of system parameters is of a subject of our future work, as it could help to decrease the acquisition time while preserving the image quality. For instance, we demonstrated^48^ that an improvement of grating quality in terms of better uniformity and higher thickness of the absorbing lines allowed to improve the visibility of the system by an additional 5–10%^48^ (in absolute values, i.e. from current 15–20% to 20–30%), which would translate into the improvement of angular sensitivity and image quality. Additionally, the maximum high voltage of the tube can be decreased from 40–50 kVp down to 20–30 kVp, reducing the number of high-energy photons, that do not contribute to the creation of contrast interference pattern. With this approach, however, one should increase the current accordingly, to make sure that the total photon flux remains and doesn’t contribute to visibility drop. Apart from this, it could be accompanied by a better thermal management of the X-ray source, which would help to increase the deposited power per squared millimeter at the target from the current value of 1 W/μm^2^, which would translate in shorter acquisition times. Finally, an optimization of the setup geometry could also take place. While being both quite compact (about 1 m in length) the TLI and MAAST setups have had different interferometric geometries (see Supplemental Material). The asymmetric arrangement of gratings of smaller periods (1 μm in G_0_ and 1.5 μm in G_1_) in the TLI system allowed to achieve better angular sensitivity and smaller effective voxel size, i.e. ultimately a better resolution. On the other hand, a symmetric arrangement of gratings with a larger period of 3 μm (aspect ratio of 23:1 in this work), allowed the MAAST setup to reach a comparable image quality and angular sensitivity. By design, symmetrical setups provide an advantage, as they reach a higher visibility and angular sensitivity for a given length and photon flux. However, the fabrication of X-ray gratings with high aspect ratio (pitch of 1 µm or even smaller, and aspect ratio of 60:1 or even higher) requires non-conventional, still under development, technologies^49^. Finally, gratings with larger periods are usually easier to align and operate, which facilitates user experience. Summarizing, all these aspects should be taken into consideration when further optimizing the parameters of the setup.

In our experiments we aimed for the highest possible resolution of breast specimen that we could achieve with GI PC X-ray imaging. Even though the best achieved resolution of 14 μm is still one order of magnitude lower than the submicron resolution achieved in the histology, GI X-ray PC lab-CT has one clear advantage—it provides pathologist with a 3D visualization of the inner sample structure. Trading spatial resolution (and acquisition time) for the sake of multimodal imaging and therefore powerful multiscale feature correlation across histological and X-ray images might be an interesting wat to explore. In the future, we plan to continue the work on reducing total acquisition time down, ideally below 1 h, while trying to maintain (and improve) resolution, contrast, and overall image quality.

In conclusion, we have explored the feasibility of using two table-top high-resolution phase-contrast CT prototypes for X-ray microscopy of breast specimen. Microscopic features, both anatomical structures, and benign and malignant calcifications could be identified and verified by histopathology. The technique has the potential to complement current histopathological workflows of breast core biopsies, by offering pathologists the opportunity to assess breast biopsies in 3D and direct their attention to calcifications and malignant structures before sectioning. It could be used as an assistance in high-resolution histopathological screening of formalin-fixed vacuum assisted biopsies (VAB), as well as for feature visualization and characterization in paraffin-embedded surgical samples as high-resolution phase-contrast imaging of soft tissue provides significantly better contrast than classical absorption.

## Methods

### Sample Preparation and Histological assessment

This retrospective ex-vivo study was conducted in accordance with the Declaration of Helsinki and was approved by the institutional review board. The use of the surgical breast samples were approved by the Cantonal Ethical Committee (KEK_2021_554) and the patients had a written informed consent to use tissue for research purposes. In total three samples (Sample 1, Sample 2, Sample 3) of approx. 3 × 3 × 3 cm^3^ each were taken from two different patients (Patient 1 and Patient 2), where there was a histologically proven invasive ductal breast cancer or intraductal carcinoma in situ (DCIS) in the postoperative sample. All samples were fixed in fixed in 4% formaldehyde. Samples were processed in the Department of Pathology and Molecular Pathology, University Hospital Zurich, Switzerland and were used for further imaging experiments. After the experiments were completed, samples were returned to the pathology department of the USZ and underwent routine embedding, processing and light microscopical examination with hematoxylin and eosin (H&E stain).

### Tomographic acquisition with asymmetric Talbot–Lau interferometric setup (TLI)

The setup was demonstrated previously^50^ and is a standard Talbot–Lau grating X-ray interferometer, consisting of a microfocus X-ray source with the size of 20 μm and a set of three gratings with small pitches, enabling the possibility of high-resolution imaging in absorption, phase-contrast and dark-field modes simultaneously. Detailed operating principles of Talbot–Lau Interferometer, layout of the setup and description of components can be found in the supplementary materials. The only modification of the setup in comparison to the previously published is a new sCMOS X-ray detector with the pixel size of 19.85 μm.

X-ray source was operated with the voltage and current of 42 kV and 1.5 mA, respectively. This resulted in the total deposited power of 63W. Gratings G0, G1 and G2 with periods of 1.0 μm, 1.5 μm, 3.0 μm, respectively, were assembled in an asymmetric setup at the 35th Talbot order and the G0-G2 distance of 80.4 cm. An angular sensitivity^51,52^ of 40 nrad and a mean visibility^53^ of 19.5% were measured^50^. The sample was placed close to the G1 grating. Considering the geometrical magnification of 3.56× due to beam divergence, the effective voxel size at the sample position resulted to be 5.57 μm.

Tomographic acquisition was done by rotating the sample over 360°, with projections acquired every 0.3°. The total number of sample data projections was 1200. Each projection was acquired with a five-position stepping scan^54^, when five images with exposure time of 50 s (for each step) were taken while the grating G2 was shifting in five steps of 0.6 μm each. To ensure the best signal-to-noise ratio, reference stepping scans without a sample were taken at every 3° and applied for the flat-field correction. Absorption, phase- and dark-field signals were retrieved from each projection according to the principles described in the supplementary materials. The total acquisition time was 91 h. and 40 min. The measured resolution of the system was measured to be 14.4 µm (supplementary Fig. S5).

### Tomographic acquisition with symmetric interferometric setup and micro-structured anode (MAAST)

Micro-focus X-ray source with a micro-array anode-structured target (MAAST) source was recently introduced by Sigray inc.^46,47^. Its key feature is the implementation of micro-structured diamond substrate with linear trenches, filled with tungsten (W). This allows to replace a combination of X-ray source and G0 by only MAAST source and potentially gain photon flux due to removal of the absorbing grating G0 from the beam path and truncation of the setup.

A detailed investigation of the performance, as well as thermal and temporal stability of the MAAST source was already published^46^. As a part of the present manuscript, we complement previous results by a direct imaging of X-ray source with a pinhole camera to demonstrate that the claimed minimal source size of 20 µm was indeed achieved. The detailed description of the experiment can be found in the supplementary materials.

The MAAST source was operated at 50 kV and 1.2 mA, providing the total deposited power of 60 W. One phase grating (G1) and one absorption grating (G2) with a period of 3 μm were placed downstream the X-ray source in the symmetric Talbot–Lau setup with the Talbot order 15 and the source-to-G2 distance of 1.088 m. Target photon energy of 20 keV was used. The same detector as for TLI was used. A mean visibility of 16% was measured and well agrees with theoretical simulations^55^ (see supplementary materials).

The sample was placed next to the G1 grating, resulting in a geometrical magnification of 2.33× and the effective voxel size of 8.52 µm at the sample position. Tomographic acquisition was performed according to the same logic as in TLI section; however, the sample was rotating over 185° with acquiring a projection every 0.25°. The total number of sample data projections was 740 and this number was smaller than 1200 used for the TLI setup, as we aimed to reduce the total acquisition time. Reference stepping scans for flat-field correction were taken at every 5°. For the Sample 2, the dataset with exposure time per image of 60 s was recorded, resulting in the total acquisition time of 64 h. 45 min. The angular sensitivity of 62 nrad was also measured. For the sample 3, the datasets with different exposure times were recorded, resulting in different total acquisition times (Table 1). The measured resolution of the system was measured to be 18 µm (supplementary Fig. S6).

**Table 1:** Exposure time per image and its relation to the total acquisition time per one CT scan of the Sample 3.

Exposure time per image, s	Total acquisition time, min	Approx. total acquisition time, h
60	3885	65
30	1943	32
15	971	16
10	648	11
5	324	5
1	65	1

One projection was formed by five images (i.e. the stepping scan). 777 total projections (sample data and flatfield acquisitions, 3885 images) per scan were acquired.

### Tomographic Reconstruction, Image Processing and Resolution

Tomographic reconstructions were done using the Astra toolbox package^56–58^. For the phase-contrast CT reconstructions, the built-in BP3D_CUDA algorithm and Hilbert filtering with default settings were used. For the absorption-contrast CT reconstruction, FDK_CUDA with the standard filter was used. For visualization purposes, phase-contrast X-ray images presented in the manuscript were processed using non-linear contrast enhancement method (CLAHE), implemented in Fiji 2.3.0/1.53f^59^ with the following parameters: blocksize = 127, histogram bins = 256, maximum slope = 2. Any additional filtering is mentioned in figure captions. 2D image resolution was assessed by the Fourier Ring Correlation (FRC)^60^ (see supplementary Figs. S5 and S6 for more details).

### Deep learning-assisted denoising

A U-net with 7 million parameters has been used to investigate the feasibility of data-driven denoising of our images. Given the absence of clean ground truth data, the model has been trained in a Noise2Noise fashion^61^. In particular, the network has been trained to map a noisy image with shorter exposure time to an image with longer exposure time, e.g. 30–60 s. Both images were reconstructed with filtered back projection (FBP) in conjunction with the Hilbert filter. The network was trained on an independent sample and later applied to unseen data. In particular, the training and validation batches were composed of randomly cropped parts of the larger tomography. Despite the fact that we used a single sample to train the network, we were able to achieve good performance on the unseen data due to the very large data size of the tomography used for training. The Adam optimizer^62^ was used for training together with batch normalization and L2 regularization.

### Resolution measurement with the Fourier ring correlation

The resolution of the tomographic data acquired with TLI and MAAST setups was measured using Fourier Ring Correlation (FSC)^63–65^. The FRC yields a task-based estimate of the spatial resolution in an image by computing the correlation in the frequency space of two realizations of the same sample. For the low frequencies there will always be a near-perfect correlation. Towards the higher frequencies the correlation decreases until it approaches zero when there is no detectable signal left. In our case, it has been calculated between two volumes obtained by splitting the available set of angular projections in half. A fixed threshold of 0.5 was used for the evaluation of the resolution at the FRC curve. One should consider this resolution highly dependent on the image contrast, provided by the content of the volume of a real sample in the presence of some characteristic features. Therefore, the provided numbers should be perceived as the estimation of the resolution for the breast samples used in the present manuscript, and not as the limiting value of the system in general. Moreover, the resolution was estimated with the twice reduced angular step of CT the scan. It should thus be better at the real measurement due to the fact that an angular undersampling decreases the resolution radially, which can be observed in the graphs of Figs. S5 and S6.

## Supplementary Information

Supplementary Information.

## Data Availability

The datasets generated during and/or analyzed during the current study are available from the corresponding author on request.
