# Supplementary material for: Towards virtual histology with X-ray grating interferometry
Source: Sci Rep. 2023 Jun 3;13:9049. doi: 10.1038/s41598-023-35854-6 (PMC10239461; doi:10.1038/s41598-023-35854-6)
Supplement: Supplementary file 1 — Supplementary Information. [file 41598_2023_35854_MOESM1_ESM.docx]

# Supplementary Materials

**Operation Principle of a Talbot-Lau Interferometer**

A key benefit of the Grating Interferometer (GI) is the possibility to restore absorption, phase-contrast and dark-field signals, revealing absorption, refraction and scattering features inside the sample, respectively^56^. GI is based on the Talbot effect, when a periodic phase grating (G1) is exposed to spatially coherent X-ray radiation, which creates an interference pattern, representing the image of the G1 at the given Talbot distance^65^.

First of all, to make the Talbot effect even possible, spatial coherence length should be equal or larger than the grating period. Spatial coherence length is measured at the G1 plane and is proportional to the ratio 2 $\frac{}{s}d$, where is X-ray wavelength, *s* is the size of an X-ray source and *d* is the distance between the source and G1. This implies limitation on the size of the X-ray source as it has to be small, which in its turn also decreases the generated photon flux. To preserve some flux, one can keep a larger source and simultaneously achieve high spatial coherence at the G1 plane, if absorption grating G0 with similar or even smaller period (than G1) is placed next to the X-ray source. Thus, G0 forms a secondary photon source, which fulfills the above equation with *s* being equal to the period of G0.

The fractional Talbot distance of the order *m*, produced by the π-shift G1 grating (used in the present manuscript), can be calculated by the formula:

$D_{m}=m*\frac{{p_{1}}^{2}}{8\lambda}$ (1)

where *m* is the Talbot order (*m* = 1,3,5…) and *p_1_* is the period of G1.

Talbot pattern is formed at the aforementioned distance, and changes when the sample is placed on the beam path. To record μm-scale shifts of the Talbot pattern, a detector with a comparable pixel size of a few μm would be needed. However, one typically tries to increase photon efficiency by utilization of X-ray detection systems with larger pixel sizes. Therefore, an analyzer grating G2 can be used, being placed at the certain distance, *d*, from G1.

For the case of the cone-beam X-ray setup, one can define G0-G1 distance, *l*, G1-G2 distance, *d*, period of G2, *p_2_* and period of G0, *p_0_* , accordingly:

$d=MD_{m}$; $l= \frac{M}{M-1} D_{m}$; $\frac{p_{0}}{p_{2}}=\frac{l}{d}$; $M=\frac{l+d}{l}$ (2)

The period of the Talbot pattern is half the period of the modulating grating G1 (for π-shift G1). For two setups, described in the present manuscript, it means that one can place G2 at the distance $d=2D_{m}$ (M=2, *p_2_=p_1_=p_0_* , *d=l*, symmetric setup) or $d=4D_{m}$ (M=4, p_2_ = 2p_1_ = 3p_0_ , *d = 3l*, asymmetric setup).

G2 grating can be displaced along its transverse direction over one period with small steps and thus allows a detector with larger pixel sizes to record several images with intensity modulations. This technique is called stepping scan and in the present manuscript, it was formed by five consecutive displacements of 0.6 μm over one period of G2 grating (3 μm). In order to correct for background variations when revealing sample contrast, two acquisitions are done – one with and one without the sample. Intensity signal in each point of the G2-plane oscillates as the function of the displacement during the stepping scan, therefore a difference of phases of two curves in two measurements (with and without the sample) in each pixel is related to the phase profile of the object^56^ . The mean intensity of the curve in its turn is related to the absorption in the object. Altogether, this allows to reconstruct the absorption and phase-contrast radiographic projections.

**Performance of X-ray systems and Image Quality Assessment**

Performance of a Talbot-Lau Interferometer is assessed with two parameters – grating visibility, V, and angular sensitivity $\alpha_{min}$. Visibility is measured as the ratio of the amplitude over the average intensity of a phase stepping curve. This value typically is of the range 15-30%, strongly depending on grating quality and Talbot order.

Angular sensitivity is the smallest refraction angle that still can be resolved by the system. It serves as a performance metric for phase-contrast imaging systems and can be defined by the formula $\alpha_{min}= \frac{L_{G1}}{L_{s}}\frac{p_{2}}{2\pi d}\sigma_{\varphi}$, where L_G1_ and L_s_ are distances from the X-ray source to the G1 grating and to the sample, respectively; p_2_ and d is the period of the G2 grating and G1-G2 inter-grating distance, respectively; $\sigma_{\varphi}$ is the standard deviation (noise) of phase shift measurement, which can be measured at reconstructed differential phase-contrast image in the area without a sample. It has been demonstrated^52,66^ that angular sensitivity of 100 nrad or less is desirable for the high-contrast imaging of the biological soft tissue imaging.

**Description of the TLI setup**

**
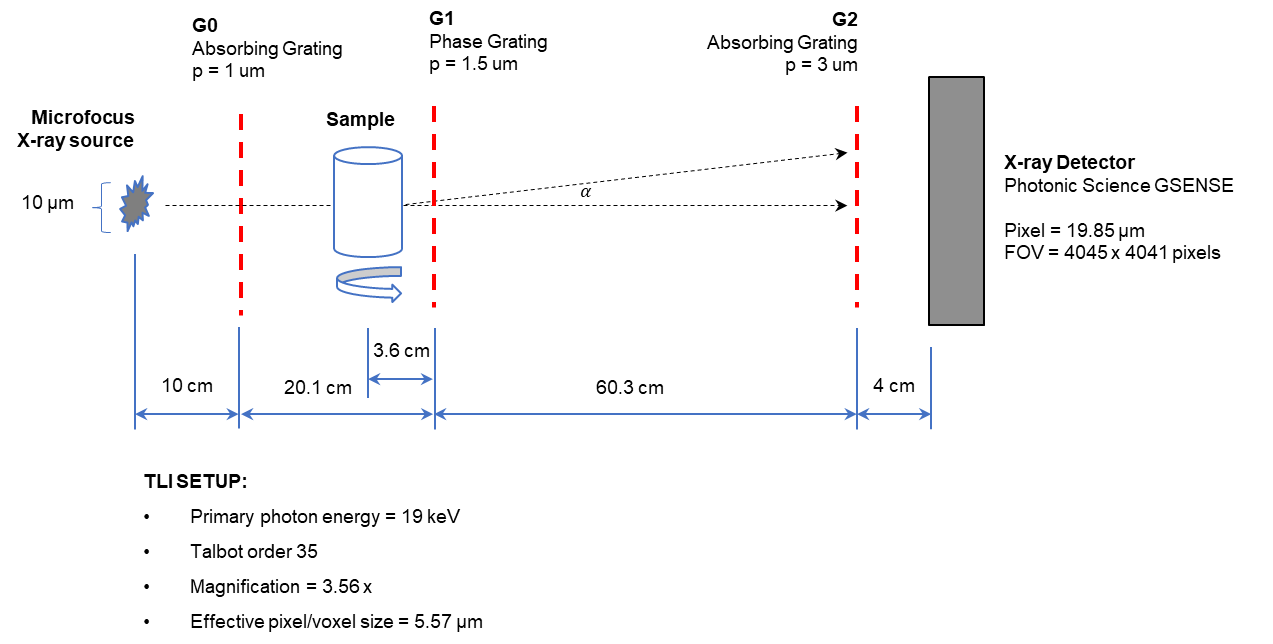
**

**Supplementary Figure S1.** Schematic representation of the Talbot-Lau Interferometer (TLI) setup.

TLI setup:

- Microfocus X-ray source with a tungsten target (Sigray inc.); source size of 10 μm;
- X-ray gratings (production details were previously published^52^): G0 is the linear absorbing grating with the period of 1 μm and the thickness of 30 μm, produced by combining optical lithography, deep reactive ion etching of silicon and atomic layer deposition of iridium. G1 is the linear pi-shifting phase grating with the period of 1.5 μm and the thickness of 25 μm, produced by deep reactive ion etching^67^. G2 is the linear absorbing grating with the period of 3 μm and the thickness from 30 μm to 35 μm, produced by deep silicon reactive ion etching and filled with gold by seedless conformal electroplating^68^. Gratings were assembled in the asymmetric setup at the 35^th^ Talbot order and the G0-G2 distance of 80.4 cm, to achieve higher angular sensitivity.
- The sample was mounted on a motorized stage (HUBER Diffraktionstechnik GmbH), equipped with two tilts and one axial rotation stages, allowing for an accurate sample positioning and rotation during the tomographic acquisitions.
- X-ray sCMOS detector – Photonic Science X-ray GSENSE 16.4 MP ([link](https://photonicscience.com/products/x-ray-cameras/x-ray-scmos-16mp-detector/) to the product description), pixel size of 19.85 μm, field of view (FOV) of 4045 × 4041 pixel^2^ or approximately 8 × 8 cm^2^. Taking magnification into account, this translates into the maximal sample size of 2.2 × 2.2 cm^2^

**Description of the MAAST setup**


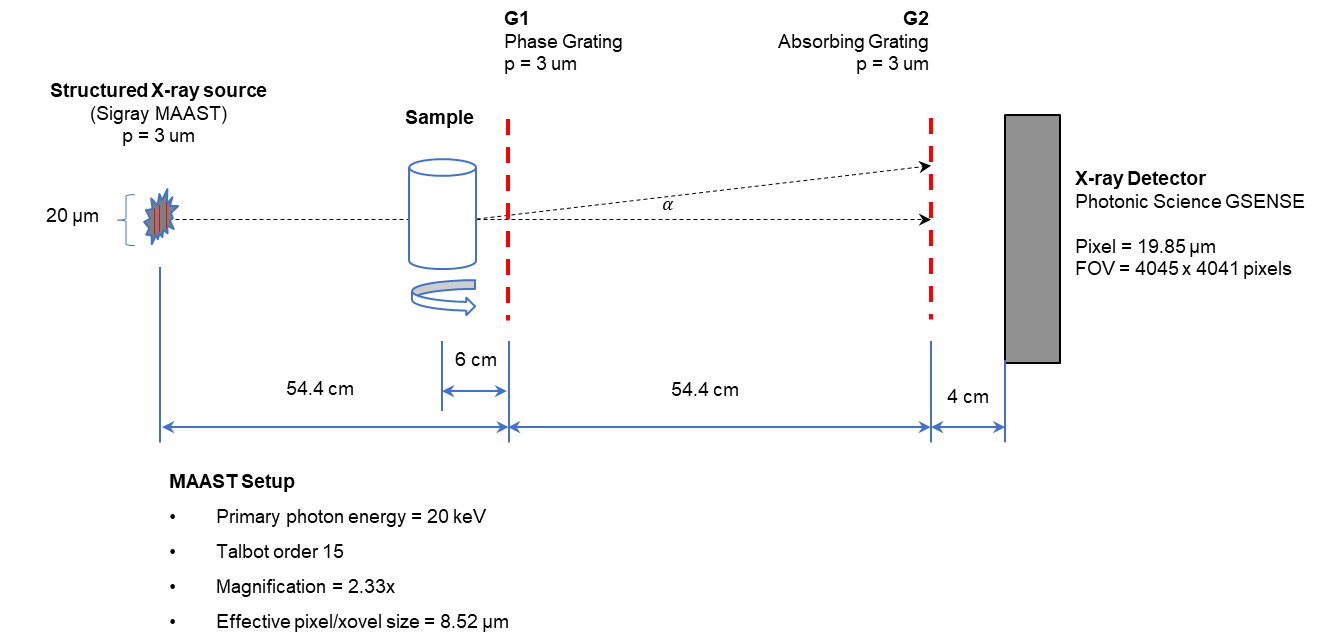


**Supplementary Figure S2.** Schematic representation of the MAAST setup.

MAAST setup:

- Microfocus X-ray source with a structured anode (Sigray inc.), source size of 20 μm;
- X-ray gratings: G1 is a pi-shifting linear phase grating produced by deep reactive ion etching of silicon. It has the period of 3 μm and the height of 25.5 μm. G2 is the linear absorbing grating produced by deep reactive ion etching of silicon^67^ and filled with gold by seedless conformal electroplating^68^. It has the period of 3 μm and the height from 30 μm to 35 μm. Gratings were assembled in the symmetric setup at the 15^th^ Talbot order and the source-to-G2 distance of 108.8 cm to achieve higher angular sensitivity.
- The sample was mounted on a motorized stage (HUBER Diffraktionstechnik GmbH), equipped with two tilts and one axial rotation stages, allowing for an accurate sample positioning and rotation during the tomographic acquisitions.
- Photonic Science X-ray GSENSE 16.4 MP X-ray detector, with a 4045 × 4041 pixel^2^ sCMOS sensor and a pixel size of 9 μm. Structured 100 μm Csl scintillator coupled with a fiber optic with a 2.2:1 demagnification ratio. This results in the effective pixel size of 19.85 μm, providing the effective field of view (FOV) of approximately 8 × 8 cm^2^. Taking X-ray magnification of the MAAST setup into account, this translates into the maximal sample size of 3.5 × 3.5 cm^2^.

**MAAST – source size**

One important parameter that define the resolution of the X-ray system is the size of the photon source, *s*. A bigger source size affects the resolution of the laboratory tomographic system by the simple equation $\frac{\text{Δ}\text{ }}{s}=\frac{L_{2}}{L_{1}}$, where *L_1_* and *L_2_* are source-to-sample and sample-to-detector distances, respectively, while parameter *Δ* defines the image blur, occurring at the detector plane. Therefore, *Δ* should be smaller than, at least, the double of the pixel size (a typical resolution of X-ray detectors), which translates into the numerical requirement *Δ < 40 µm* for the MAAST setup and the detector described in the present paper.

To confirm the size of the X-ray source, generated by the Sigray MAAST tube, we made a direct imaging of the photon source with the pinhole, which follows the principle of the camera obscura. For this test (supplementary Fig. S3), a 5 μm pinhole was located between the source and the X-ray detector such, that 48x magnified image of the source was recorded with the detector. Source size is highly dependent on two operating voltages inside the MAAST source: G1 and G3, which are responsible for the selection of the appropriate electron emission area on the cathode, and for the focusing of the electron beam at the anode, respectively. Tuning G1 and G3 allowed to vary the size of the MAAST source and achieve its minimal size of 20 µm at G1 = 20V, G3 = 1050V. As mentioned in the paragraph above, this value is sufficient for providing the high-resolution imaging with no blurring by the source.


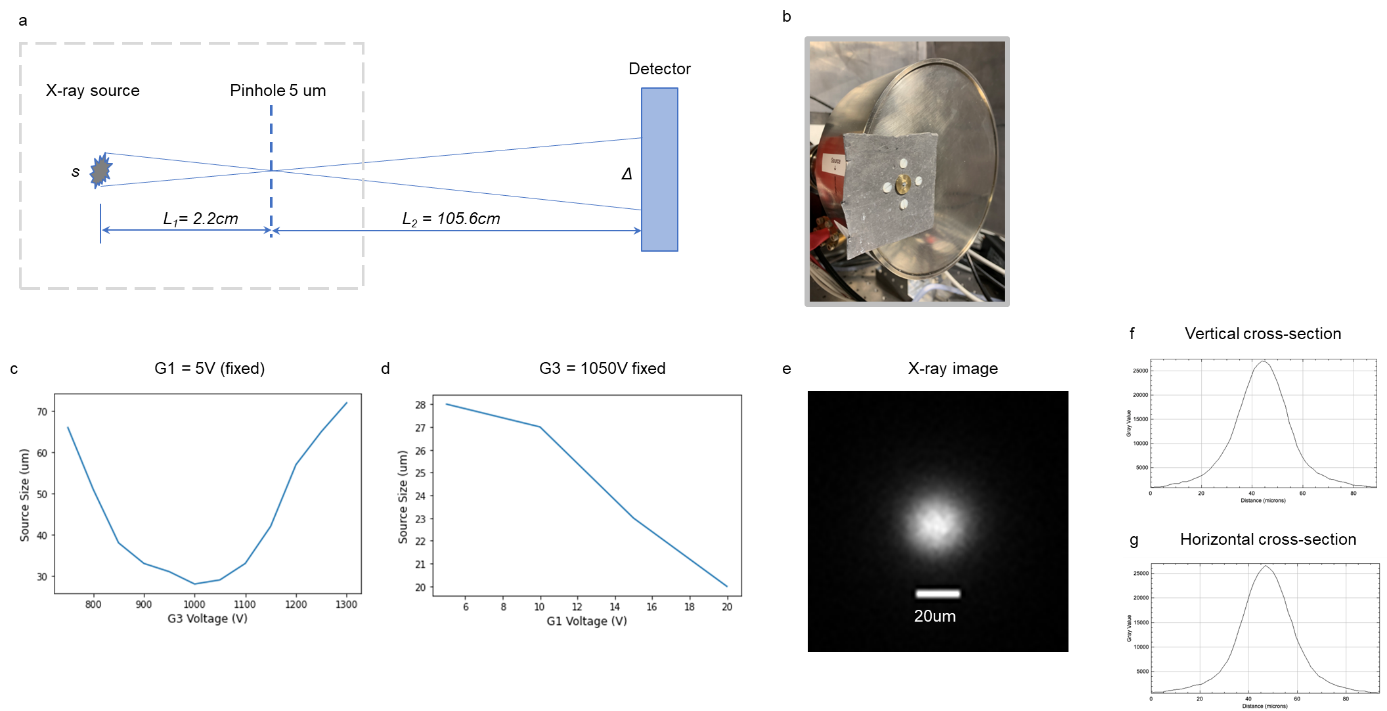


**Supplementary Figure S3.** Experimental measurement of the size of the Sigray MAAST X-ray source. (*a*) - The layout of the experiment with the direct imaging of the source by the pinhole. (*b*) - The photo with the part of the setup in gray dashed box on *a*. (*c*) - Dependence of the source size on the G3 voltage, which is responsible for anode focusing of the electron beam. G1 is fixed at 5V; (*d*) - Dependence of the source size on the G1 voltage, which is responsible for cathode emission. G3 is fixed at 1050V; (*e*) – X-ray image with the minimal size of the source that was achieved at G1 = 20V, G3 = 1050V. Central cross-sections in vertical (*f*) and horizontal (*g*) directions demonstrate symmetrical and well-pronounced profiles with full widths at half maximum of 20 μm.

**MAAST – measured and calculated visibility**

**
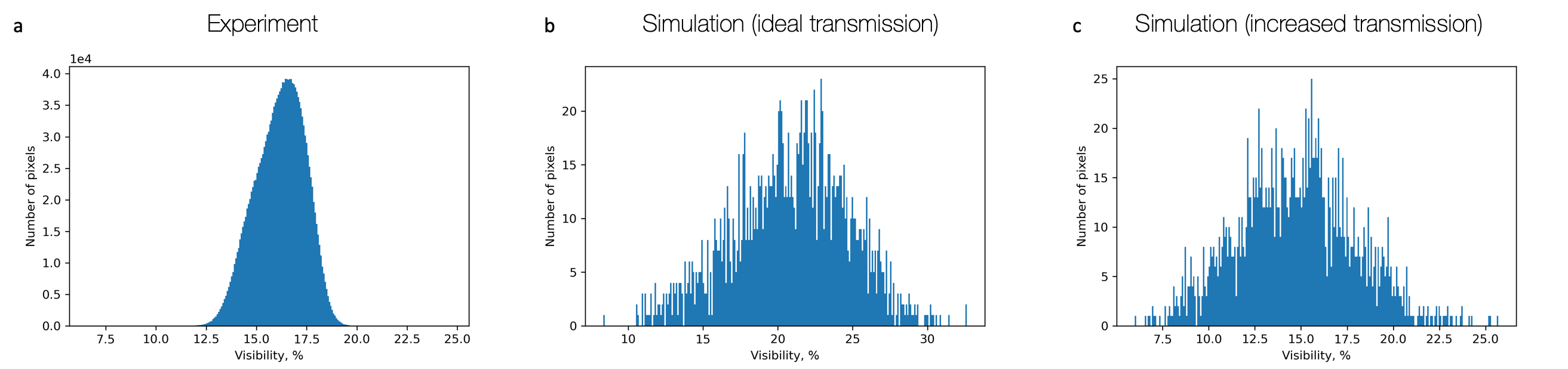
**

**Supplementary Figure S4.** Experimentally measured and calculated visibility of the MAAST setup. (*a*) The histogram with pixel distribution of the experimentally recorded visibility at the MAAST setup. The region of interest corresponds to the position of the sample. The mean visibility of 16% and peak visibility of 25% are observed at the uniform visibility distribution curve. (*b*) EGS_XGI^57^ Monte-Carlo (MC) simulation of the MAAST setup results in the ideal mean visibility of 21% that could be measured at the detector position. For this case, the assumption of the ideal emittance of the structured anode is used: 100% emitting slits interchanged with perfectly absorbing slabs (0% emittance) of the anode. (*c*) Simulation with a non-ideal emittance model of the structured anode (by increasing the 0% emittance of the slabs to 13.5%) reduces the mean visibility to 16%. Increasing the emittance between the active regions is a first step towards accounting for the more complex inner structure, the non-uniformity of the anode and potential scattering radiation. However, apart from the simplified source model the MC assumes ideal flat gratings and perfectly parallel grating alignment. This leads to an expected overestimation of the visibility, e.g., seen by the high visibility bins in the histograms from both simulations. Therefore, even if an average emittance of 13.5% between the line sources might be a bit high, the MC results indicate that the maximally achievable visibility with ideal gratings is between 16% and 21% dependent on the quality of the source shape, which agrees well with the experimental findings.

**Resolution measurement with the Fourier Ring Correlation**

**Supplementary Figure S5.** Resolution measurement for the TLI setup. (a) One of two 2D slices (Sample 1) that were generated from two statistically independent tomographic reconstructions with twice decreased angular step: exposure of 50 sec/projection, step of 0.6 deg/projection (used for FRC), coverage of 360 deg. (b) As the resolution may depend on the distance between the observation point and the rotation axis, it is measured in different regions with sampling by 70 × 70 pixel^2^ area. Minimal resolution of 14.4 µm was observed at the rotation axis.

**Supplementary Figure S6.** Resolution measurement for the MAAST setup. (a) The one of two 2D slices (Sample 2) that were generated from two statistically independent tomographic reconstructions with twice decreased angular step: exposure of 60 s/projection, step of 0.5 deg/projection (used for FRC), coverage of 185 deg. As the resolution may depend on the distance between the observation point and the rotation axis, it is measured in different regions with sampling by 70 × 70 pixel^2^ area. Minimal resolution of 18 µm is observed at such maps - both for the datasets with the exposure time of 60 s per image and 5 s per image.

**Binning and reduced angular step**


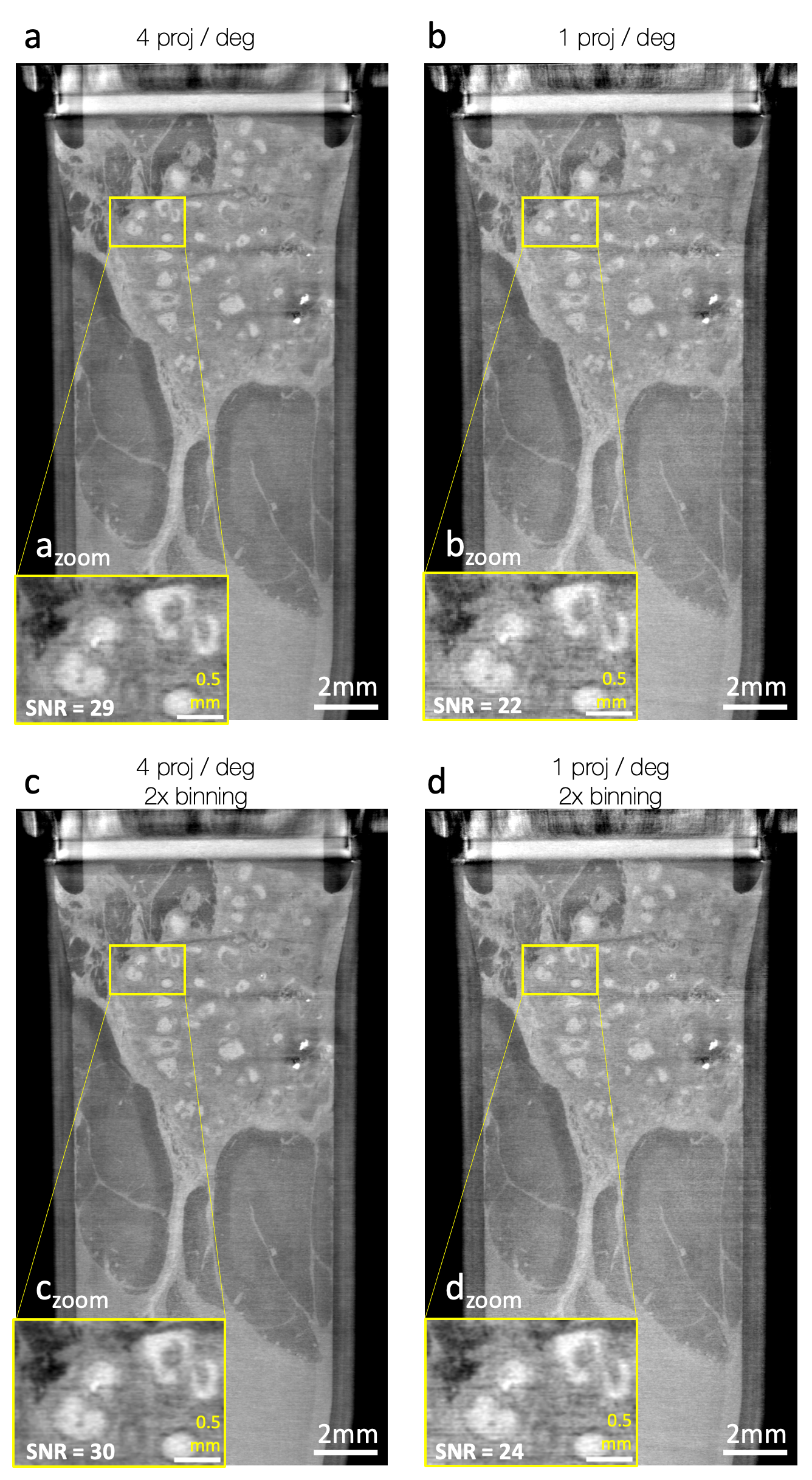


**Supplementary Figure S7.** Different reconstruction modes for the sample 3 illustrate the influence of the used number of projections per degree and of binning on the image quality of the tomogram. Phase-contrast; 60-sec acquisition; coronal tomographic slice, identical (but not the same) to what has been shown in fig. 4 (b). (a) Data reconstruction with 4 projections per degree (every 0.25°), full resolution, as described in the MAAST methods section of the main body of the paper. (b) Data reconstruction with 1 projection per degree (every 1°), full resolution. (c) Data reconstruction with 4 projections per degree (every 0.25°), and twice binned pixel size. (d) Data reconstruction with 1 projection per degree (every 1°), and twice binned pixel size. (a_zoom_ - d_zoom_) zoomed sections (yellow square box) of each corresponding image show that the reduction of the number of projections per degree leads to the increased image noise and gradual reduction of the resolution in the axial direction, nevertheless allowing to reduce the absorbed dose (4x in this case). Subsequent binning helps to slightly reduce the noise, however decreases the resolution, proportionally to the binning factor (2x in this case).
